# Supplementary material for: Suppression of Pro-Inflammatory M1 Polarization of LPS-Stimulated RAW 264.7 Macrophage Cells by Fucoxanthin-Rich Sargassum hemiphyllum
Source: Mar Drugs. 2023 Oct 12;21(10):533. doi: 10.3390/md21100533 (PMC10608208; doi:10.3390/md21100533)
Supplement: Supplementary file 1 [file marinedrugs-21-00533-s001.zip › marinedrugs-2655152-supplementary.pdf]

Supplementary information

Supplementary Figure S1.

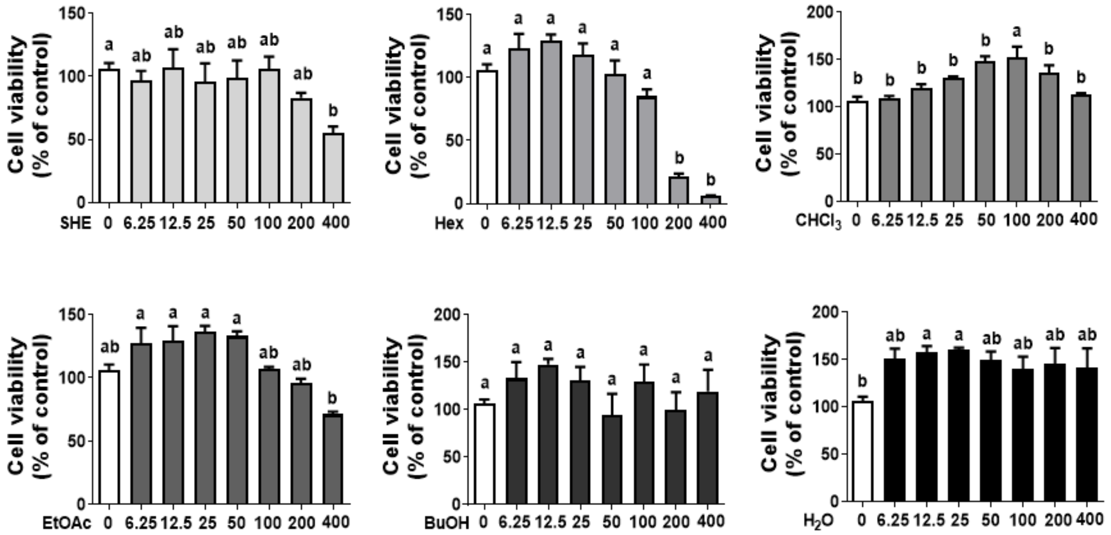

Cytotoxicity of SHE and fractions on RAW 264.7 macrophages. RAW 264.7 cells exposed to the indicated concentrations of SHE and different fractions for 24 h, and cell viability was measured at 570 nm using the MTT assay. Treatment concentrations of each sample were 0, 6.25, 12.5, 25, 50, 100, 200, 400 μg/mL. Different letters on the point indicate a significant difference ($p<0.05$)

Supplementary Table S1. List of the primers used in the present study.

Gene	Forward (5′→3′)	Reverse (5′→3′)
Rpl32	CAC CAG TCA GAC CGA TAT	TTC TCC GCA CCC TGT TG
Tnf	GGC TGC CCC GAC TAC GT	ACT TTC TCC TGG TAT GAG ATA GCA AAT
Il1b	GTC ACA AGA AAC CAT GGC ACA T	GCC CAT CAG AGG CAA GGA
Il6	CCC ACC AAG AAC GAT AGT CA	CTC CGA CTT GTG AAG TGG TA
Cox2	GCC TAC TAC AAG TGT TTC TTT TTG CA	CAT TTT GTT TGA TTG TTC ACA CCA T
Nos2	AAT CTT GGA GCG AGT TGT GG	CAG GAA GTA GGT GAG GGC TTG
Cd86	ACG ATG GAC CCC AGA TGC ACC A	GCG TCT CCA CGG AAA CAG CA
Ccl2	GGC AGA GAA GCA TGG CCC AGA A	CTT CTG GGC CTG CTG TTC A
Cat	CAC ACA CAC ACA CAT GCA ATA C	TTC TGA GTG GGC CAT CTT TAT C
Sod1	GAG ACC TGG GCA ATG TGA T	GTT TAC TGC GCA ATC CCA CT
Nef2l2	CTC GCT GGA AAA AGA AGT G	CCG TCC AGG AGT TCA GAG G
Gpx1	GTC CAC CGT GTA TGC CTT CT	TCT GCA GAT CGT TCA TCT CG
Nox1	TTC ACA GTT ATT CAT ATC ATT GC	AGA GAA CAG AAG CGA GAG
Cybb	CCC TTT GGT ACA GCC AGT GAA GAT	CAA TCC CGG CTC CCA CTA ACA TCA
Hmox1	CTC TCT TCT CTT GGG CCT CTA A	TGT CAG GTA TCT CCC TCC ATT C

[1] Abbreviations used: Rpl32, Ribosomal protein L32; Tnf, Tumor necrosis factor-α; Il1b, Interleukin-1β; Il6, Interleukin 6; Cox2, Cyclooxygenases2; Nos2, inducible nitric oxide synthase; Cd86, Cluster of Differentiation 86; Ccl2, chemokine (C-C motif) ligand 2; Cat, Catalase; Sod1, Superoxide dismutase 1; Nfe2l2, Nuclear factor erythroid-2-related factor 2; Gpx1, Glutathione Peroxidase 1; Nox1, NADPH oxidase 1; Cybb, Cytochrome b-245 beta polypeptide isoform 1; Hmox1, Heme Oxygenase 1.
